# Forecasting the carbon footprint of civil buildings under different floor area growth trends and varying energy supply methods

**DOI:** 10.1038/s41598-023-49270-3

**Published:** 2023-12-11

**Authors:** Jiaying Teng, Hang Yin

**Affiliations:** School of Economics and Management, Jilin Jianzhu University, Changchun, 130118 China

**Keywords:** Environmental impact, Urban ecology

## Abstract

The energy consumption and carbon footprint of buildings are significantly impacted by variations in building area and the number of households. Therefore, it is crucial to forecast the growth trend of building area and number of households. A validated time series model is used to predict the new building area in Jilin Province from 2023 to 2030. The new building area in Jilin Province is expected to exhibit two trends of growth in the future: rapid growth (S1) and slow growth (S2). By 2030, under the S1 growth trend, the residential construction area and public building construction area in Jilin Province are expected to be 30.26 Mm^2^ (million square meters) and 7.23 Mm^2^, respectively. If the future floor area grows slowly under the S2 trend, the new floor area of different types will be 8.26 Mm^2^ and 1.33 Mm^2^ by 2030, respectively. The population growth shows a downward trend. Therefore, the energy consumption and carbon footprint of new buildings with different growth trends of floor areas and the number of households can be predicted. The energy consumption of new buildings shows an increasing trend from 0.32 Mtce in 2023 to 0.55 Mtce in 2030 under the S1 trend and a slight downward trend under the S2 trend. The carbon footprint is expected to be reduced by 0.017–0.311 million tons of CO_2_ when using heat pumps to supply 10–50% of the heat and wind and solar to supply 10–50% of the electricity. For every 10% increase in the use of ultra-low energy buildings, the energy consumption of civil buildings decreases in the range of 0.0063–0.028 Mtce. If the use of heat pumps and renewable energy increases by 10%, the energy consumption of civil buildings decreases in the range of 0.0054–0.0249 Mtce.

## Introduction

Energy consumption in buildings escalated from 115 EJ in 2010 to approximately 135 EJ in 2021, accounting for nearly 30% of global energy consumption. Indirect carbon emissions, resulting from the consumption of electricity and heating in building operations, constituted 19% of the total carbon emissions from the energy sector^1^.

Global governments have enacted policies to foster energy efficiency within the building sector^2^. The European Commission has instituted a threshold system predicated on energy performance to evaluate the contribution of renewable and district cooling towards EU renewable energy objectives^3^. In Canada, the government has launched the Community Buildings Retrofit Initiatives to promote retrofits that enhance energy performance and curtail emissions in community buildings^4^. China’s 14th 5-Year Plan for Building Energy Conservation and Green Building Development is designed to augment heating system efficiency and achieve a 30% improvement in the energy efficiency of residential buildings by 2025^5^.

The fluctuation in building area and household count exerts a substantial impact on the carbon footprint of buildings. Huo et al.^6,7^ utilized the Monte Carlo simulation methodology to predict the influence of China’s urbanization rate on building carbon emissions. Findings suggested that under a business-as-usual scenario, the energy peak for the rural residential building sector will be attained first in 2027. Nonetheless, the impact of the urbanization process on the future peak of building carbon emissions remains an area of uncertainty. A sensitivity analysis conducted by Ma et al.^8^ demonstrated that per capita floor space and energy intensity of urban residential buildings exert the most significant influence on emission peak uncertainty. The energy consumption and carbon footprint of buildings are profoundly affected by fluctuations in building area and household count. This study conducted by Ahn and Sohn^9^ confirmed that the energy consumption of multi-family housings in Seattle is influenced by urban form variables such as horizontal compactness, vertical density, and variation of building heights, and is also spatially dependent. Timuçin Harputlugil et al.^10^ proved that the activities conducted by occupants within a building significantly influence its energy consumption. Consequently, it is of paramount importance to predict the growth trajectory of building area and number of households.

As building area and household count fluctuate over time, classical regression models are unable to accurately forecast their progression. However, time series methods can utilize historical data to predict future developments for these variables^11^.The Auto-regressive Integrated Moving Average (ARIMA) model is a time series forecasting method that is widely used in the field of civil engineering to predict future values of a variable based on its past values. The Seasonal Auto-regressive Integrated Moving Average (SARIMA) model is an extension of the ARIMA model that can handle seasonal patterns in the data. The objective of time series analysis is to derive significant statistical insights and attributes from the data and utilize them to forecast future values^12^. Ngo et al.^13^ formulated a model to forecast the energy usage in buildings for the upcoming day, utilizing the SARIMA method. In the case of predicting civil construction area and residential households, the ARIMA/SARIMA model is often used because it is a simple and effective method that can capture the trends, seasonality, and auto-correlation in the data^14^.

The way energy is supplied has a notable impact on the energy consumption of buildings. The utilization of air source heat pumps can conserve energy while simultaneously satisfying the augmented demand for health, thermal comfort, and productive indoor environments^15^. Buffa et al.^16^ investigated 40 operational thermal networks in Europe using distributed heat pumps, which can aid in de-carbonizing the heating and cooling sector and utilize low-temperature heat sources. These networks can also operate at close-to-ground temperatures. Ren et al.^17^ carried out a study is to enhance the performance of a hybrid system that combines cooling, heating, and power by utilizing natural gas, solar, and geothermal energy resources. The research indicates that optimizing the variable output ratio of the ground source heat pump and operating the system using the following electric load strategy leads to the optimal performance of the system. Elnabawi et al.^18^ evaluated the use of hybrid system in an educational building which can provide up to 23% energy savings annually, and the integration of photovoltaics can further reduce the building’s energy consumption by almost 85% and significantly cut down carbon emissions.

Although the share of modern renewable totaled 10.1% in 2019 in the heating sector, burning biomass for heating still represented more than 30% of total renewable energy use^19^. Heat pumps serve as a crucial component in tackling climate change by providing an alternative to gas- and oil-fired boilers. Nevertheless, it is essential that heat pumps are powered by low-emission electricity to significantly reduce greenhouse gas emissions compared to conventional heating equipment^20^. Promoting environmentally-friendly consumption patterns and increasing the penetration of cleaner energies in urban households would effectively mitigate the rise in building carbon emissions^21^.

In essence, the pursuit of energy efficiency and carbon reduction transcends the mere integration of energy-conserving technologies; it necessitates a comprehensive strategy that encompasses a multitude of elements. Consequently, this manuscript delves into the following areas of interest:

1. Utilizing time series forecasting methodologies to predict the expansion trajectory of building areas and household counts.
2. Examining the carbon footprint of buildings during operation under varying trends of new floor area growth.
3. Evaluating the impact of different proportions of renewable energy and ultra-low energy buildings on the trend of carbon footprint and the energy consumption of buildings throughout their operational lifespan.

The findings of this manuscript serve as a predictive tool for the growth trends of carbon footprints in buildings, and as a basis for proposing solutions aimed at carbon reduction.

## Methodology

### Research scheme

In this paper, the Jilin province of China (an ASHRAE 6A climate zone) is used as an example to carry out the study. Jilin province is located in the northeastern part of China and is known for its long and cold winters. The province has been used as an example to develop energy-efficient building designs that can withstand the harsh winter conditions and reduce energy consumption^22^.

**Figure 1:**
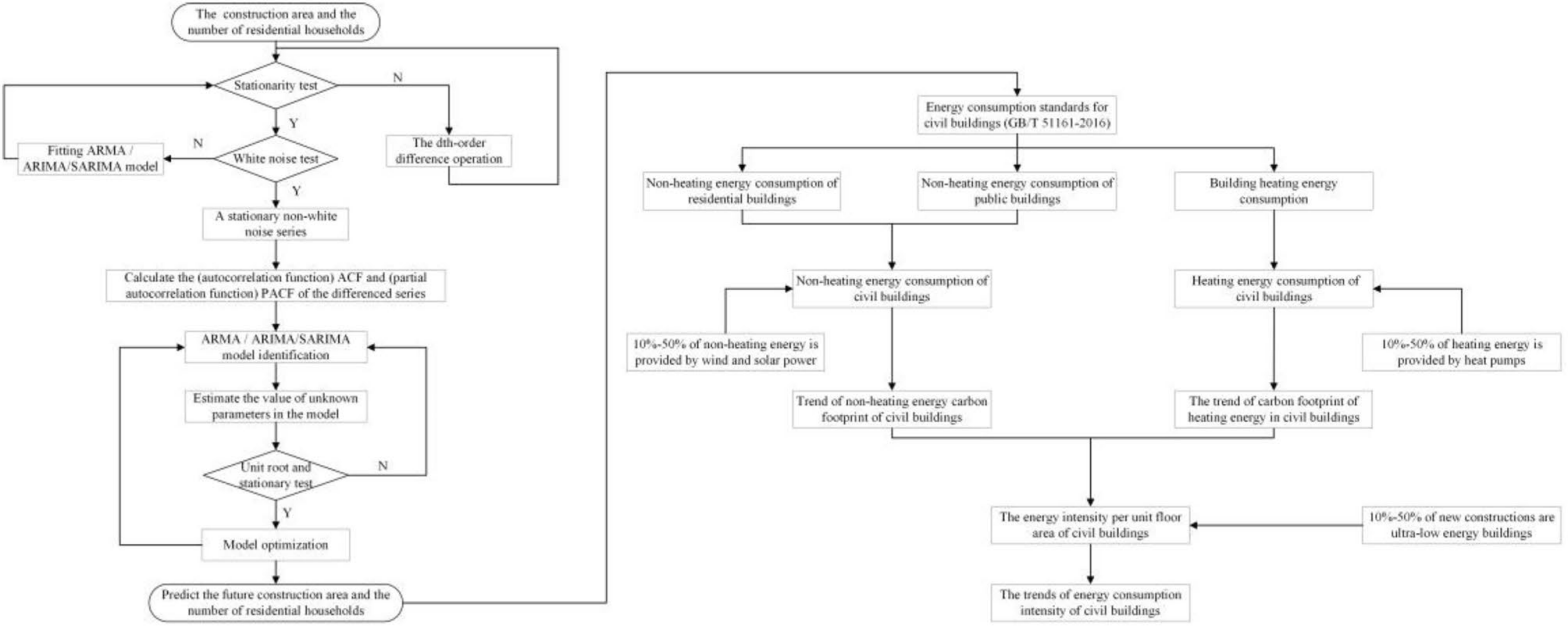
Research scheme.

The research scheme of the study is shown in Fig. 1.

1. An ARIMA/SARIMA model is established to predict the annual new civil construction area and the number of residential households through the time series method. Civil buildings consist of residential buildings and public buildings.
2. The heating and non-heating energy consumption of new civil buildings are calculated separately according to the energy consumption standards for civil buildings of China (GB/T 51161-2016).
3. The tendency of carbon footprint of new civil buildings is analyzed as 10–50% of heating energy is provided by heat pumps and 10–50% of non-heating energy is provided by wind and solar power.
4. The energy intensity of new civil buildings after utilizing renewable energy is calculated. The trends of energy consumption intensity of new civil buildings are analyzed when 10–50% of new constructions are ultra-low energy buildings.

### Prediction model generation based on time series method

An SARIMA (Seasonal Autoregressive Integrated Moving Average)^23^ model is established based on the data of new construction area in Jilin Province from 2001 to 2022, as shown in Eq. (1).

1 $$\begin{array}{c}\left\{\begin{array}{c}\begin{array}{c}{\varphi }_{p}\left(B\right){\Phi }_{P}\left({B}^{s}\right){\nabla }^{d}{{\nabla }_{s}^{D}x}_{t}={\theta }_{q}\left(B\right){\Theta }_{Q}\left({B}^{s}\right){\delta }_{t}\\ {\varphi }_{p}\left(B\right)=1-{\varphi }_{1}B-\dots -{\varphi }_{p}{B}^{p}\\ {\Phi }_{P}\left({B}^{s}\right)=1-{\Phi }_{1s}{B}^{s}-\dots -{\Phi }_{Ps}{B}^{Ps}\\ {\nabla }^{d}={\left(1-B\right)}^{d}\\ {\nabla }_{s}^{D}={\left(1-{B}^{s}\right)}^{D}\end{array}\\ {\theta }_{q}\left(B\right)=1-{\theta }_{1}B-\dots -{\theta }_{q}{B}^{q}\\ {\Theta }_{Q}\left({B}^{s}\right)=1-{\Theta }_{Qs}{B}^{s}-\dots -{\Theta }_{Qs}{B}^{Qs}\end{array}\right.\end{array}$$

where B represents lag operators, ${\varphi }_{p}\left(B\right)$ is the autoregressive polynomial, ${\Phi }_{P}\left({B}^{s}\right)$ is the seasonal autoregressive polynomial, ${\theta }_{q}\left(B\right)$ defines the moving average polynomial, ${\Theta }_{Q}\left({B}^{s}\right)$ defines the seasonal moving average polynomial, ${x}_{t}$ is the data series of the new construction area and ${\delta }_{t}$ shows the random perturbation sequences.

The total number of households in Jilin province from 1990 to 2020 is obtained from the China Population and Employment Statistics Yearbook^24^. The ARIMA model is built using the stats models toolkit in python, and 90% of the data set is taken as the training set and 10% of the data set is taken as the test set. This split ratio ensures that the model has sufficient data to learn from and that there is enough data left for testing. A larger training set can lead to better model performance, but it can also increase the risk of overfitting, where the model becomes too complex and fits the training data too closely, resulting in poor generalization to new data. The model’s performance is evaluated on a representative sample of the data by using a smaller test set, which can help to minimize overfitting and ensure the model’s generalizability^25^.

The Bayesian information criterion (BIC) is used to select the best-fit model and predict the trend of the number of residential households between 2021 and 2030. The BIC is a sound approach because it is effective at selecting simpler models when the sample size is small relative to the number of parameters in the model. The period between 2021 and 2030 was chosen for forecasting because it is a reasonable time frame (5–10 years) for predicting future trends in civil construction area and residential households. The underlying trends during this time frame would be stable and there would be no major disruptions or changes in the data that would affect the accuracy of the forecasts^26^.

### Calculation of building energy consumption

Building energy consumption is calculated as shown in Eq. (2).

2 $$ E_{b} = E_{rnh} + E_{pnh} + E_{h} $$

where ${E}_{b}$ is building energy consumption (kgce, kgce stands for kilograms of standard coal equivalent), ${E}_{rnh}$ stands for non-heating energy consumption of residential buildings (kgce); ${E}_{pnh}$ represents non-heating energy consumption of public buildings (kgce), and ${E}_{h}$ is building heating energy consumption (kgce).

Non-heating energy consumption of residential buildings is calculated based on Eq. (3).

3 $${E}_{rnh}=\left[\left({E}_{e}*H*{\lambda }_{e}+{E}_{g}*H*{\lambda }_{g}\right)*{N}_{H}/\left({S}_{p}*P\right)\right]*{S}_{r}$$

where ${E}_{e}$ is the comprehensive electricity consumption (kWh/household, including electricity consumed by air conditioning, ventilation, lighting, domestic hot water, and elevator), H stands for the number of residential households, ${E}_{g}$ represents the non-heating natural gas consumption (m^3^/household), ${\lambda }_{e}$ is the conversion coefficient of electricity to standard coal and ${\lambda }_{g}$ is the conversion coefficient of gas to standard coal. Besides, ${N}_{H}$ stands for the average number of people per household, ${S}_{p}$ is the housing area per capita (m^2^), $P$ represents the number of population and ${S}_{r}$ equals to new residential building area (m^2^).

Non-heating energy consumption of public buildings is calculated according to Eq. (4).

4 $${E}_{pnh}=\left({S}_{a}*{E}_{a}+{S}_{c}*{E}_{c}+{S}_{o}*{E}_{o}\right)*{\lambda }_{e}$$

where ${S}_{a}$, ${S}_{c}$, and ${S}_{o}$ are office building areas, commercial building areas, and other non-industrial building areas, respectively (m^2^). ${E}_{a}$, ${E}_{c}$, and ${E}_{o}$ stand for non-heating energy consumption of office building, commercial building, and other non-industrial building, respectively (kWh/m^2^).

Building heating energy consumption can be obtained by Eq. (5).

5 $${E}_{h}=S*E*\lambda +S*{E}_{p}*\left(1-\eta \right)*{\lambda }_{e}$$

where $S$ is the civil building area (m^2^), $E$ represents fuel consumption for heating (kg/m^2^), $\lambda$ stands for the conversion coefficient of fuel to standard coal, ${E}_{p}$ is the electricity consumption of pumps in the heating system (kWh/m^2^) and $\eta$ is the heating loss rate in the heating system (%).

### Carbon footprint

The carbon footprint of fossil energy^27^ is measured as follows:

6 $$CF=\sum_{i}{F}_{i}*{NCV}_{i}*{CC}_{i}*{OF}_{i}*44/12$$

where $CF$ is the carbon footprint of fossil energy (t CO_2_), $F$ represents the consumption of the fossil energy (t or 10^4^ Nm^3^), $NCV$ defines the lower heating value (GJ/t or GJ/10^4^ Nm^3^), $CC$ shows the carbon content per unit calorific value (t C/t or t C/10^4^ Nm^3^), $OF$ is the carbon oxidation rates of fuels (%), and $44/12$ defines the conversion factor of carbon to CO_2_. Besides, the subscript i represents the i-th fossil energy.

The carbon footprint of non-fossil energy sources^28^ can be computed as below:

7 $${E}_{j}=\sum_{j}{AD}_{j}*{EF}_{j}$$

where $E$ is the carbon footprint of non-fossil energy (t CO_2_), $AD$ defines the consumption of non-fossil energy (MWh) and $EF$ is the non-fossil energy carbon emission factor (t CO_2_/MWh). Besides, the subscript j represents the j-th non-fossil energy.

The computation of the carbon footprint generated by heat pumps (${E}_{e}$, t CO_2_) is derived by the following method:

8 $${E}_{e}=\frac{{C}_{e}}{SCOP}*{EF}_{e}$$

where ${C}_{e}/SCOP$ is the power consumption of heat pumps (kWh), and ${EF}_{e}$ defines the grid carbon emission factors (t CO_2_/kWh).

## Results and discussions

### Forecast of new civil building area and number of residential households

#### Civil building area

**Figure 2:**
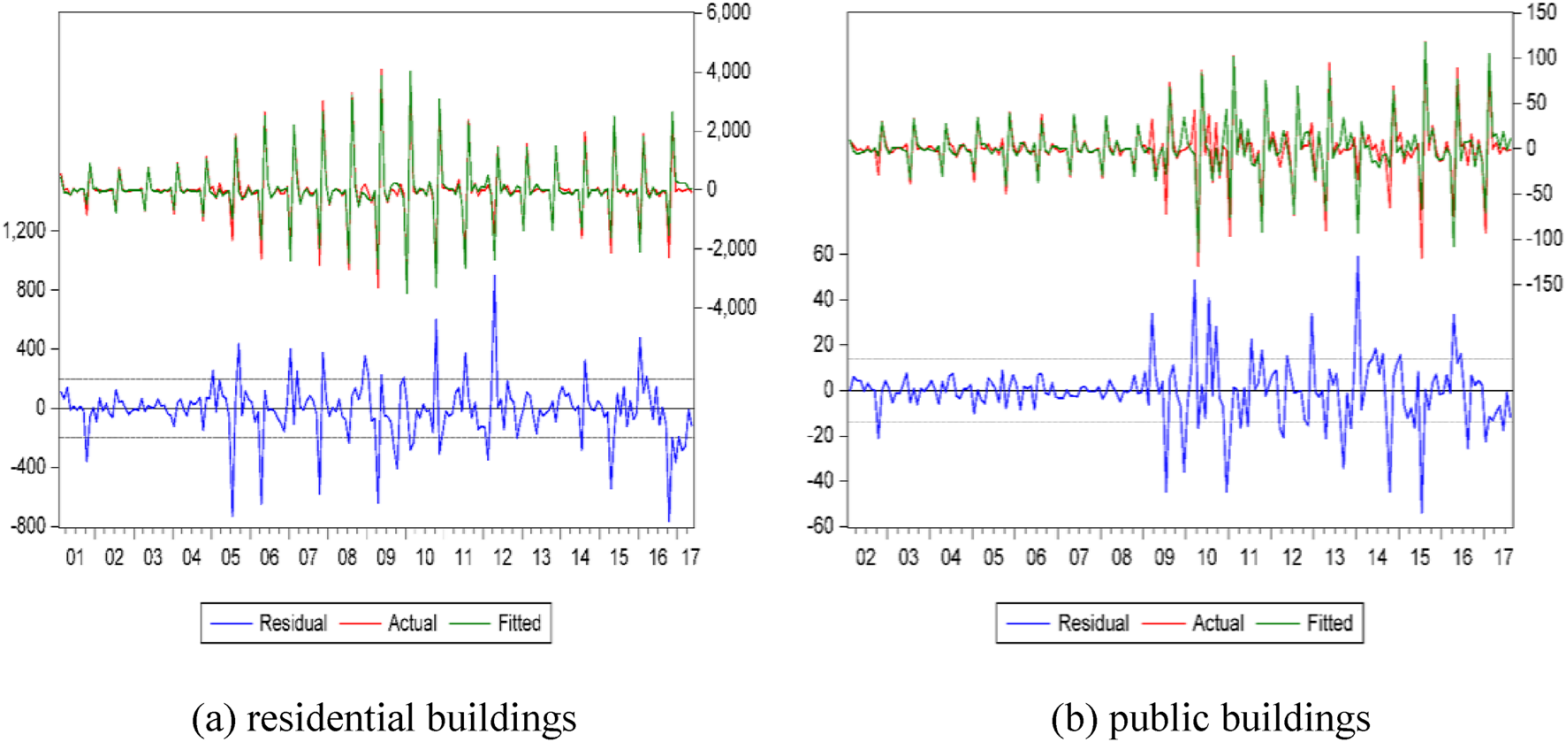
The comparison of the actual construction area with the predicted construction area.

The prediction model is first validated by comparing the new construction area simulated by the SARIMA model with the actual new construction area. The prediction model and model accuracy of new construction area in Jilin Province are shown in Table 1, and the data fitting profiles are illustrated in Fig. 2 and Table 2. As shown in Fig. 2, it can be found that the time series method has a high accuracy and can be used for the prediction of future construction area changes by comparing the actual construction area with the predicted (or fitted) construction area from 2001 to 2022.

**Table 1:** The prediction model of new construction area in Jilin Province.

Building type	Time series model	Model Accuracy	
		R^2^	BIC
residential buildings	SARIMA(2,1,1)(0,1,0)_12_	0.973	10.135
public buildings	SARIMA(2,0,0)(2,1,0)_12_	0.950	4.057
total area of new constructions	SARIMA(2,1,1)(0,1,0)_12_	0.972	10.672

**Table 2:** The fitting degree of the prediction model.

	Residential buildings	Public buildings	Total area of new constructions
R^2^	0..973	0.950	0.972
RMSE	146.179	6.449	190.192
MAPE	138.173	711.655	93.228
MAE	103.469	4.693	133.768
BIC	10.160	4.057	10.672

**Figure 3:**
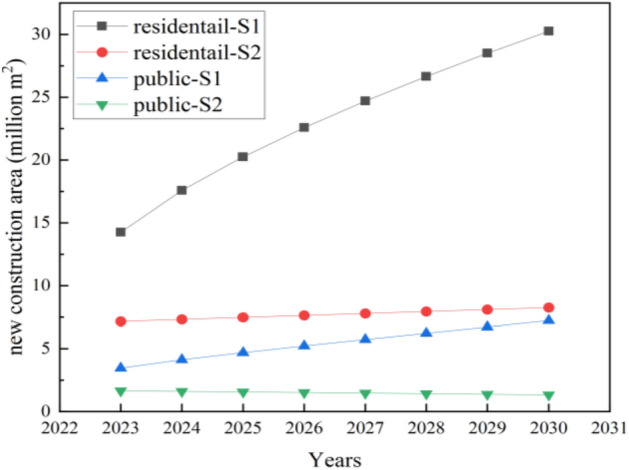
The new construction area of different types of buildings in Jilin Province.

Therefore, the time series model in Table 1 is used to predict the new building area in Jilin Province from 2023 to 2030, and the results are shown in Fig. 3. The new building area will exhibit two trends of growth in the future: rapid growth (S1) and slow growth (S2). By 2030, under the S1 growth trend, the residential construction area and public building construction area in Jilin Province are expected to be 30.26 Mm^2^ (million square meters) and 7.23 Mm^2^, respectively. If the future floor area grows slowly under the S2 trend, the new floor area of different types will be 8.26 Mm^2^ and 1.33 Mm^2^ by 2030, respectively. Therefore, in the subsequent study, the new construction floor area in Jilin Province is set as two development trends namely rapid growth (S1) and slow growth (S2). This concurs with the findings procured by Hong et al.^29^ They discerned that the proliferation of new construction in China is anticipated to persist through 2050, indicating an accelerated expansion trajectory. Nevertheless, enhancements in building longevity and quality, coupled with the advocacy for compact urban living, could potentially mitigate building energy consumption and associated emissions in China, thereby decelerating the growth momentum.

Several academics have incorporated a scenario for equilibrium (zero growth), alongside rapid and slow growth. Gong et al.^30^ devised three distinct scenarios to forecast the evolution of various civil building areas in China from 2020 to 2060, namely the benchmark scenario, the moderate control scenario, and the stringent control scenario. Similarly, Zhang et al.^31^ formulated three urban growth simulation scenarios spanning from 2015 to 2030, namely the historical scenario, a moderate growth scenario, and the strict restriction scenario. The velocity of urban growth under the historical growth scenario was significantly greater than the other two scenarios. Scenarios 2 and 3 were predicated on the principle of environmental conservation and exhibited decelerated urban growth patterns.

It should be noted that several external factors could be influencing these trends. For instance, market dynamics, policy changes, or technological advancements could drive periods of rapid growth (S1). Similarly, market saturation, policy constraints, or technological plateaus, could lead to periods of slow growth (S2). However, without specific information about the context of the time series data, it is challenging to definitively attribute these trends to particular external factors.

#### Number of residential households

**Figure 4:**
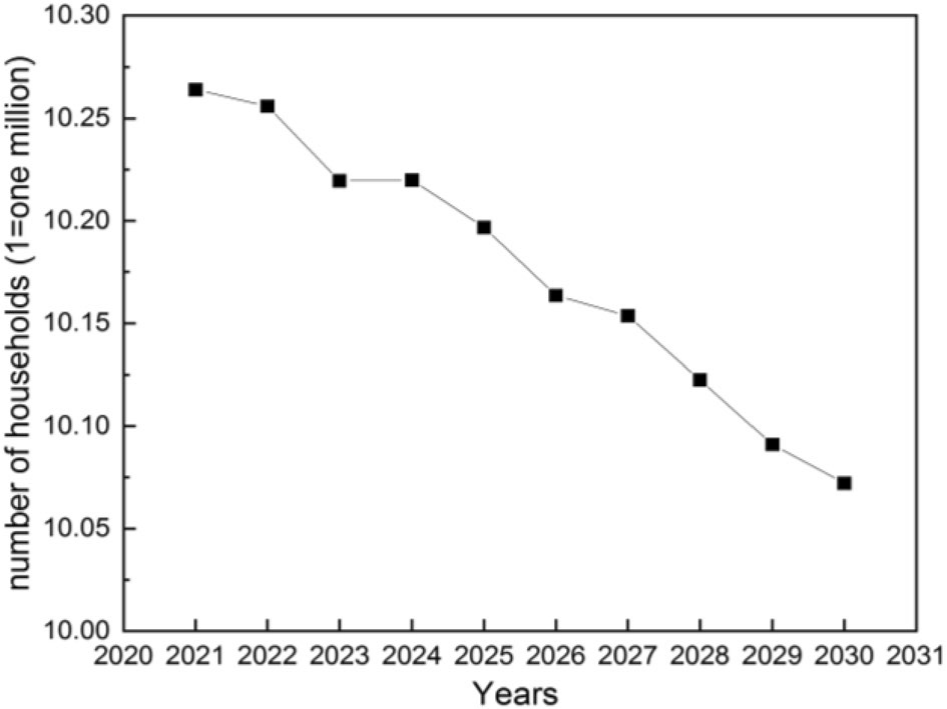
The number of residential households in Jilin Province.

The prediction results of the number of residential households are shown in Fig. 4, which decrease from 10.26 million in 2021 to 10.22 million in 2023. In 2024, the number of residential households will increase by about 360 in 2024, but still exhibits a declining trend, and in 2030, that number will drop to 10.07 million. China’s newborn population is gradually decreasing annually due to the slowdown of economic growth. At the same time, people’s willingness to give birth and get married remain low, so the population growth will show a downward trend. The number of future households will also be reduced yearly.

### Energy consumption under different new floor area growth trends

**Figure 5:**
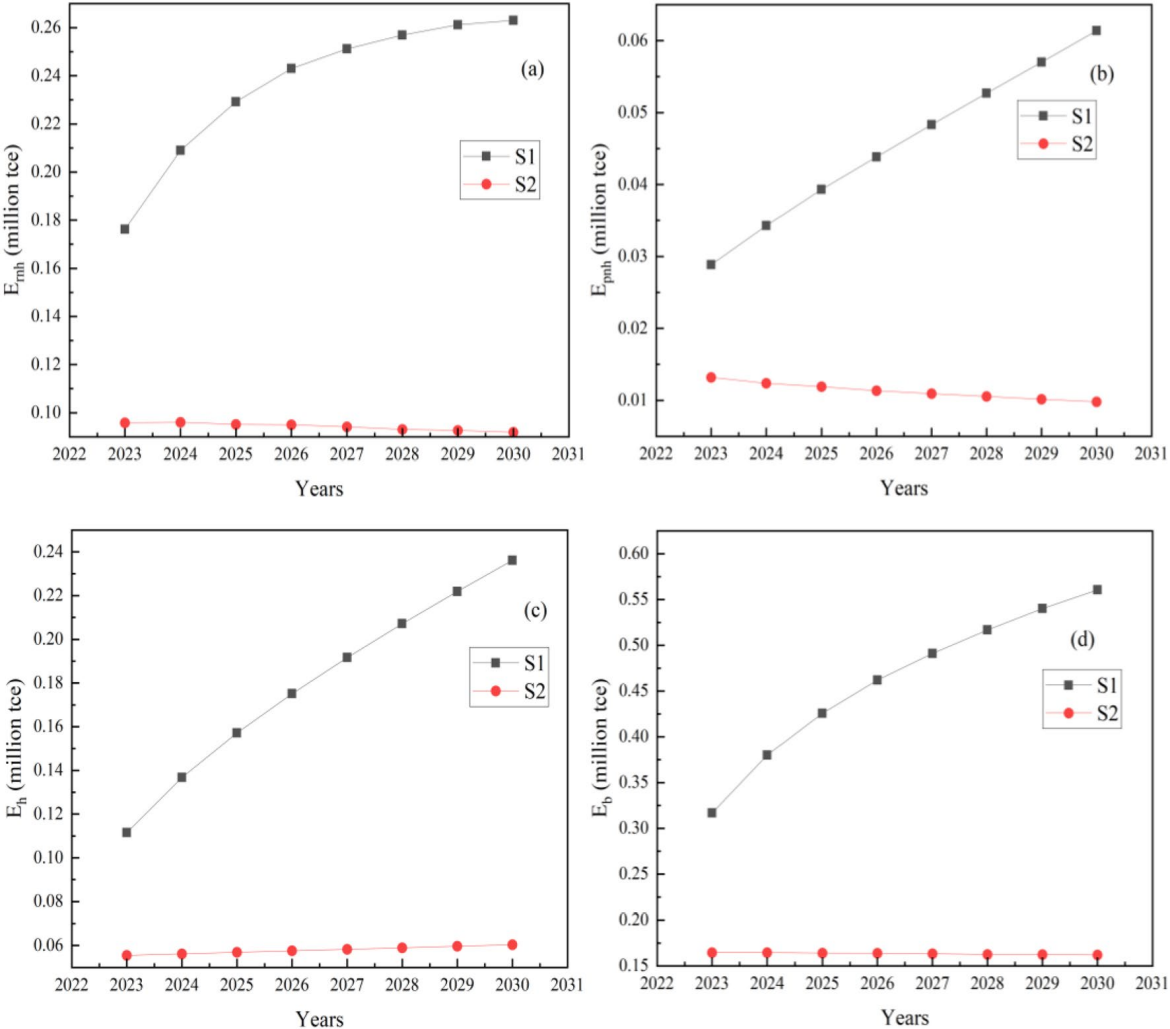
Energy consumption under different new floor area growth trends.

The energy consumption of the new constructions can be evaluated according to Eqs. (2) to (5), as shown in Fig. 5. The parameters required in the calculation are shown in Table 3. Coal is currently used as the primary heating source in Jilin Province, while several buildings also use gas and electricity for heating. Figure 5 shows the energy consumption of new buildings with different growth trends of floor areas when coal is the major heating source. The non-heating energy consumption in residential buildings (E_rnh_) mainly includes energy consumption of household appliances (such as air conditioners, lighting, refrigerators, TVs, etc.) and natural gas consumption (mainly for cooking), as illustrated in Fig. 5a. The values of E_e_ are referred to the statistical values of residential electricity demand and urban residential gas consumption indicators of major cities in Northeast China. E_rnh_ is mainly influenced by the number of residential households rather than the new floor area.

**Table 3:** The parameters required to calculate the energy consumption.

Item	${\lambda }_{e}$	$E$	$\lambda$	${E}_{p}$	$\eta$
Unit	kgce/kWh	kg/m^2^	kgce/kg	kWh/m^2^	%
Value	0.1229	6.12^a^	0.7143^b^	1.5	3

^a^The standard coal consumption for heating, and ^b^the conversion coefficient of raw coal to standard coal (obtained from the General rules for calculation of the comprehensive energy consumption GB/T 2589-2020).

Under S1 trend, E_h_ rises from 0.11 Mtce (million tons of standard coal equivalent) to 0.24 Mtce. E_h_ increases from 0.056 Mtce to 0.060 Mtce under S2 trend. E_b_ is the sum of E_rnh_, E_pnh_ and E_h_. As can be seen from Fig. 5d, under the S2 trend, E_b_ shows a slight downward trend. This is due to the more significant influence of E_rnh_ and E_pnh_ on E_b_ than that of E_h_ on E_b_ under the S2 trend.

### Carbon footprint of civil buildings during operation

Coal is currently used as the primary heating source in Jilin Province, while several buildings also use gas and electricity for heating. The city gas in Jilin Province is mainly provided by natural gas, synthetic gas and liquefied petroleum gas, accounting for about 98.45%, 1.54% and 0.01% of the total gas supply respectively, according to the data released by the National Bureau of Statistics. The parameters of coal and gas for calculating the carbon footprint are shown in Table 4. The carbon emission factor of the Northeast Regional Power Grid (${EF}_{e}$) is 0.2399 tCO_2_/MWh as published by the Ministry of Ecology and Environment.

**Table 4:** The parameters required to calculate the carbon footprint.

Fossil Fuels	${NCV}_{i}$	${CC}_{i}$	${OF}_{i}$	${\lambda }_{g}$^a^
Coal	26.7	0.03085	99	NA
Natural gas	389.31	0.01532	99	1.33
Petroleum gas	50.179	0.0172	98	1.7143
Synthetic gas	52.27	0.0122	99	0.1786

^a^The data are obtained from the General rules for calculation of the comprehensive energy consumption GB/T 2589-2020.

**Figure 6:**
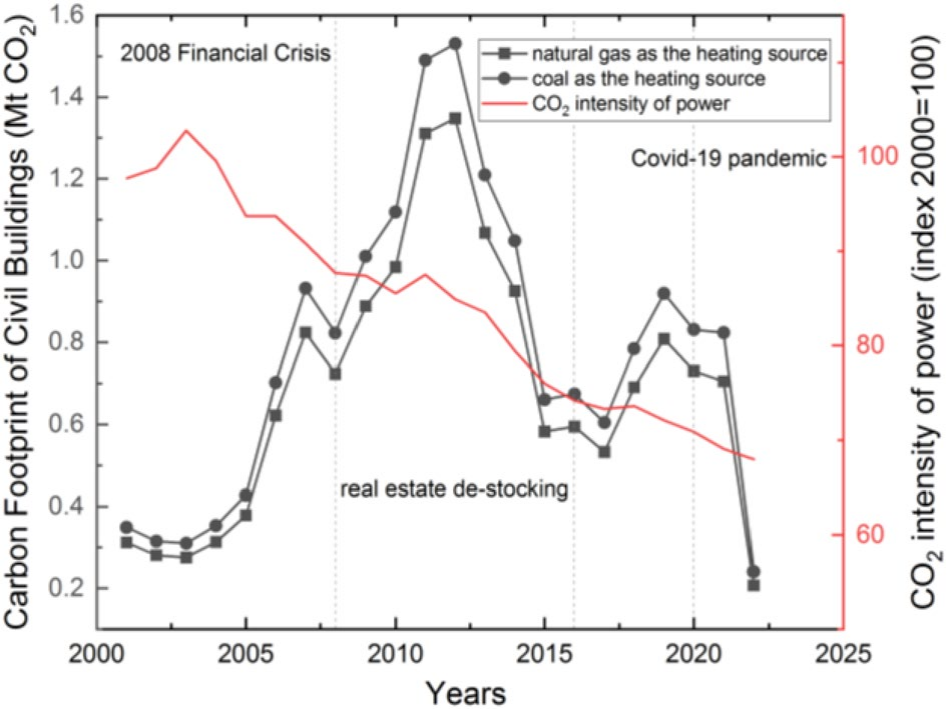
The carbon footprint of residential buildings in Jilin Province (Mt = million tons).

The carbon footprint of residential buildings in Jilin Province for the operation period from 2001 to 2022 is shown in Fig. 6. China has reduced carbon emissions from the power supply by significantly increasing the share of wind power, PV and hydropower. The coal-fired power generation percentage has decreased from 80% in 2005 to 64% in 2020. The CO_2_ intensity of power has decreased dramatically since 2003, as concluded by the international energy agency (the index 2000 = 100 means that the value of CO_2_ intensity of power in 2000 is set as 100).

However, the carbon footprint of civil buildings during the operation period is more obviously affected by the new construction area. However, the carbon footprint of civil buildings during the operation period is more obviously affected by the new construction area. From 2002 to 2008, with China’s rapid economic development, residential building area continued to increase, thus causing a continuous rise in building energy consumption. As a result of the international financial crisis, China introduced credit support and tax breaks, and the new residential building area and carbon footprint peaked in 2011–2012. In 2013–2014, China started a severe regulation of real estate, so new building construction and carbon footprint gradually decreased. In 2014–2016, China introduced a real estate de-stocking policy, which led to a renewed increase in new construction floor area and carbon footprint. After 2020, the new construction floor area and carbon footprint declined significantly due to the economic impact of the Covid-19 pandemic.

#### Carbon footprint under S1

When the new residential floor area develops with the trend of S1, the heating demand in 2030 is 10288.60 TJ, which consumes 0.185 Mtce (million tons of standard coal equivalent) or 205.77 million Nm^3^ natural gas. The electric power transmission and distribution consumption of heating supply pumps are 44.03 GWh.

**Figure 7:**
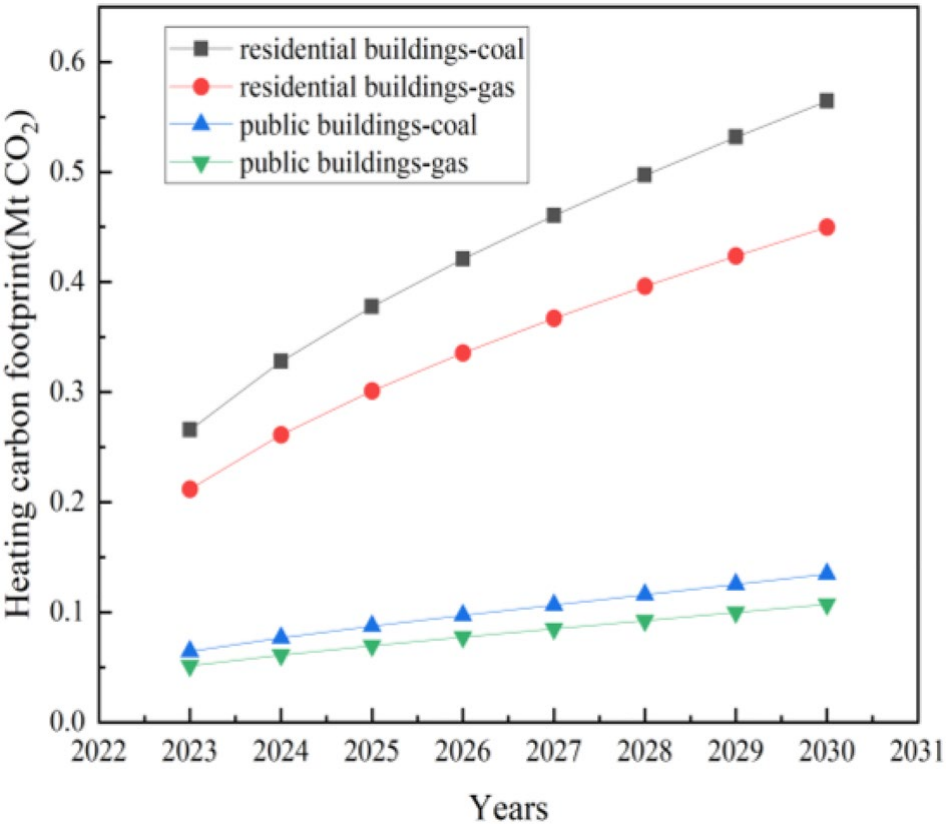
Heating carbon footprint of civil buildings.

As shown in Fig. 7, after considering the electricity consumption of heating pumps, it takes 0.19 Mtce to satisfy the heating demand, with a carbon footprint of 0. 56 Mt CO_2_. The carbon footprint of heating with natural gas is 0.11 Mt CO_2_ less than that of coal.

The heating demand for new public buildings in 2030 is 2458.20 TJ, resulting in the consumption of 0.04 Mtce or 49.16 million Nm^3^ natural gas. The carbon footprint of heating with coal increases from 0.06 Mt CO_2_ in 2023 to 0.13 Mt CO_2_ in 2030, and that of heating with natural gas increases from 0.05 Mt CO_2_ in 2023 to 0.11 Mt CO_2_ in 2030.

**Figure 8:**
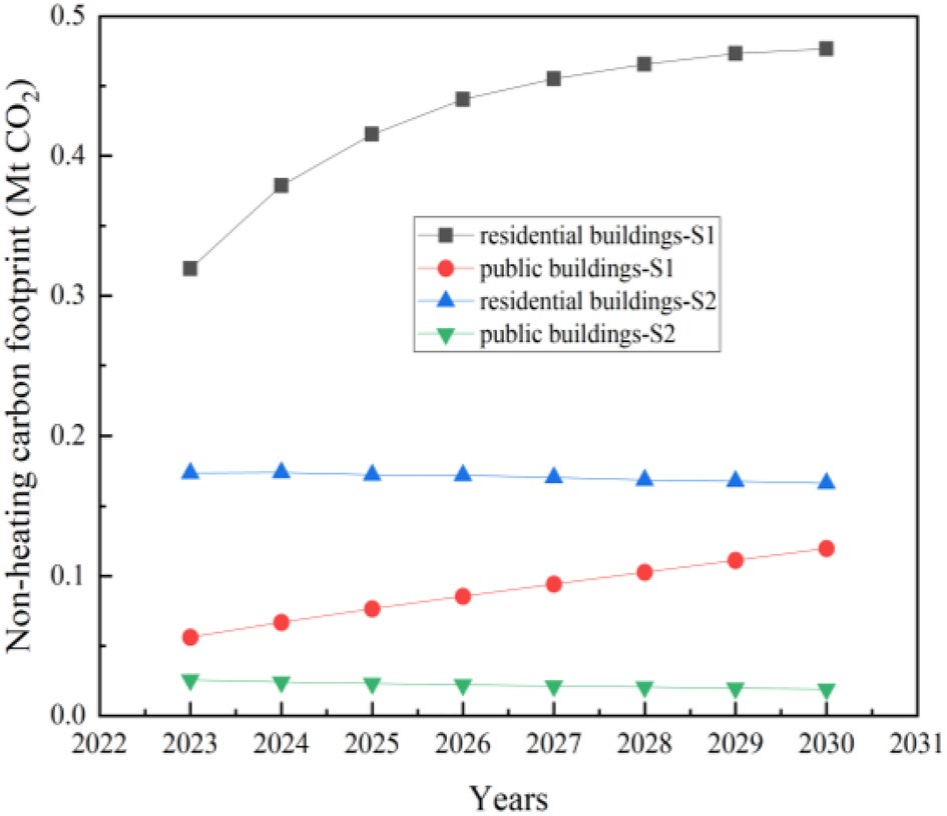
Non-heating carbon footprint of civil buildings.

The carbon footprint of non-heating energy in civil buildings mainly consists of electricity and natural gas consumption. As shown in Fig. 8, when the new residential building area increases to 30.26 Mm^2^ in 2030, it needs to consume 1231.39 GWh of electricity, and the carbon footprint will rise to 0.48 Mt CO_2_. When the new public building area grows to 2030s 7.23 Mm^2^, it will need to consume 499.33 GWh of electricity, and the carbon footprint will raise to 0.12 Mt CO_2_.

#### Carbon footprint under S2

When the new residential floor area develops with the trend of S2, the heating demand in 2030 is 2806.87 TJ, which consumes 0.05 Mtce or 56.14million Nm^3^ natural gas. The electric power transmission and distribution consumption of heating supply pumps are 12.01 GWh.

**Figure 9:**
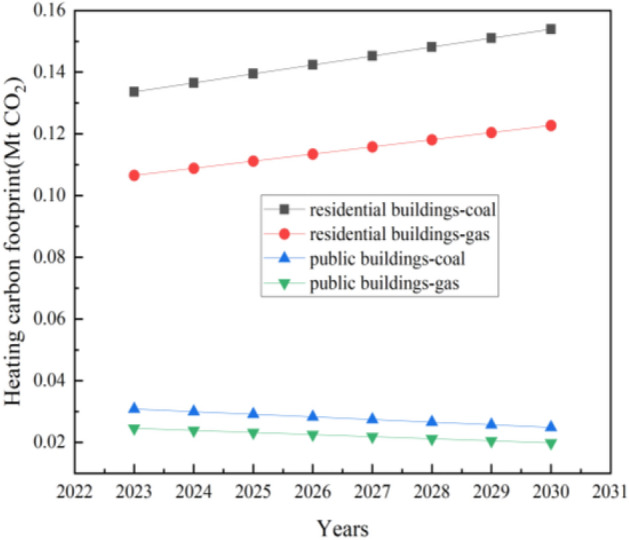
Heating carbon footprint of civil buildings under S2.

As shown in Fig. 9, after considering the electricity consumption of heating pumps, it takes 0.05 Mtce to satisfy the heating demand, with a carbon footprint of 0.15 Mt CO_2_. The carbon footprint of heating with natural gas is 0.03 Mt CO_2_ less than that of coal.

The heating demand for new public buildings in 2030 is 453.70 TJ, resulting in the consumption of 0.008 Mtce or 9.07 million Nm^3^ natural gas. The carbon footprint of heating with coal decreases from 0.03 Mt CO_2_ in 2023 to 0.02 Mt CO_2_ in 2030, and that of heating with natural gas decreases from 0.025 Mt CO_2_ in 2023 to 0.0198 Mt CO_2_ in 2030.

As shown in Fig. 8, when the new residential building area increases to 8.26 Mm^2^ in 2030, it needs to consume 430.42 GWh of electricity, and the carbon footprint will slightly decrease to 0.17 Mt CO_2_. When the new public building area decrease to 2030s 1.33 Mm^2^, it will need to consume 79.74 GWh of electricity, and the carbon footprint will decrease to 0.02 Mt CO_2_.

### Carbon footprint with renewable energy supply

China's district heating systems can be gradually changed from the current combined heating and power units (CHP) and coal-fired boilers to CHP and distributed heat pumps^32^. This paper examines the change in carbon footprint of civil buildings when using heat pumps to replace 10–50% of the heating from conventional boilers and when supplying 10–50% of the electricity from wind and solar energy. The COP (coefficient of performance) of heat pump unit is 2.3^33^, the carbon intensity of wind power is 27.48 gCO_2_/kWh^34^, and the carbon intensity of solar PV system is 92.83 gCO_2_/kWh^35^.

**Figure 10:**
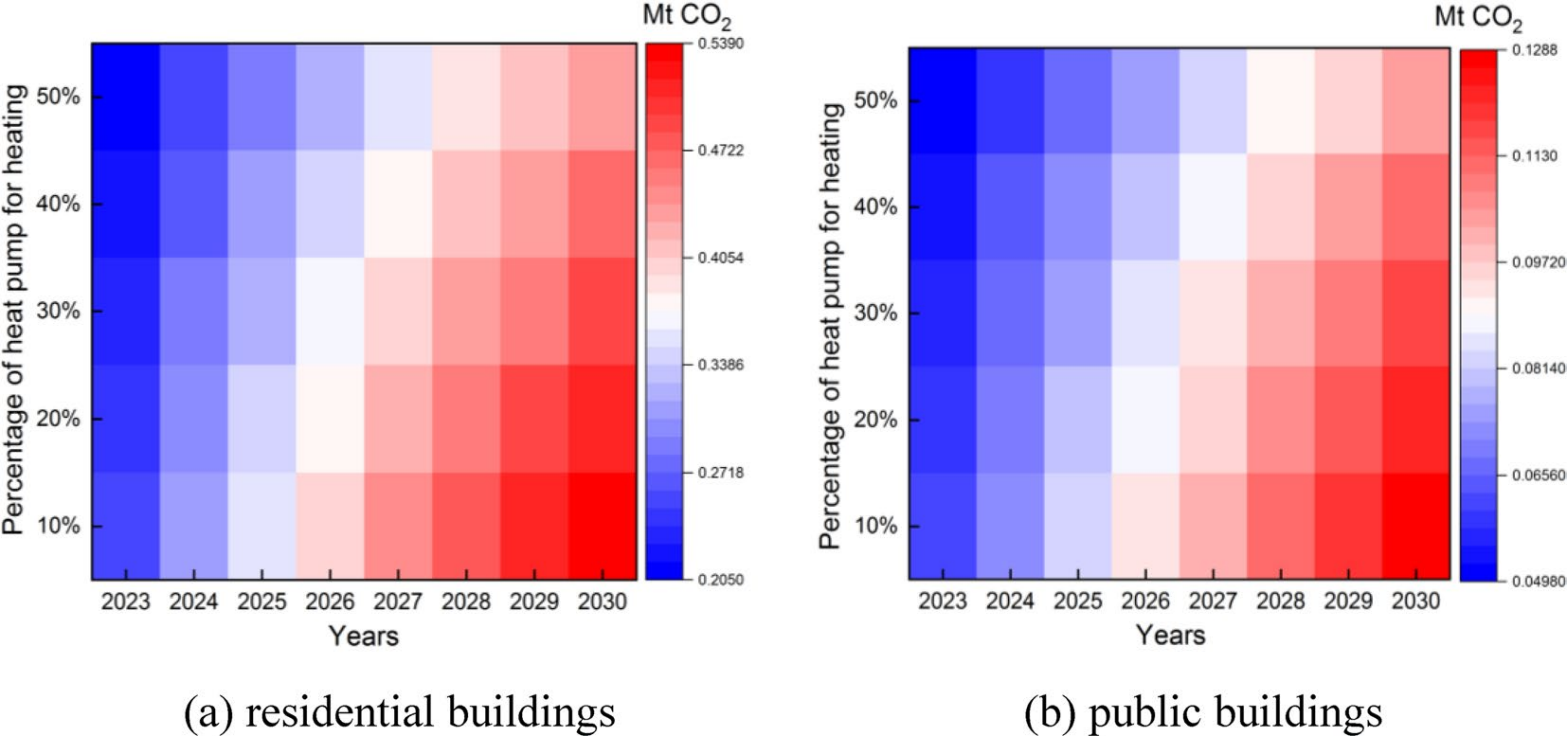
Carbon footprint with heating from heat pumps and coal.

Take S1 trend as an example. Heat pumps provide 484.38 to 5144.3 TJ of heating for residential buildings and 117.61 to 1229.1 TJ of heating for public buildings from 2023 to 2030, when 10–50% of the heat is provided by heat pumps. As shown in Fig. 10, each 10% increment in the thermal energy provided by a heat pump can reduce 0.012–0.026 Mt CO_2_ in residential buildings and 0.0029–0.0061 Mt CO_2_ in public buildings. Heat pumps consume electricity for heating, which also generates carbon emissions. For residential buildings, the total carbon emission during heating is 0.21–0.53 Mt CO_2_, and for public buildings, the total carbon emission during heating is 0.05–0.13 Mt CO_2_.

**Figure 11:**
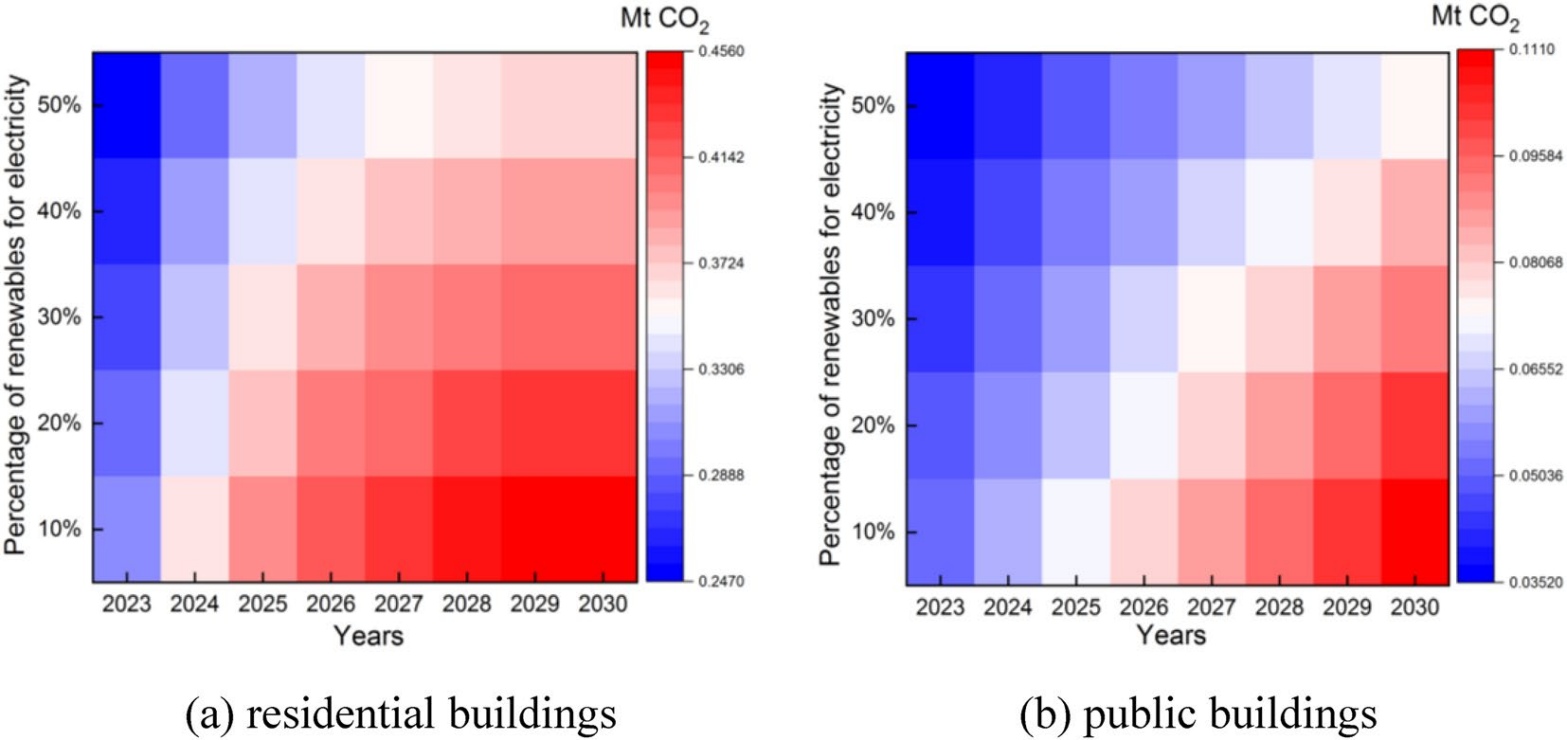
Impact of renewables’ share on carbon footprint.

Renewable energy sources (mainly wind and solar) provide 80.42 to 600.07 GWh of electricity for residential buildings and 23.47 to 249.66 GWh of electricity for public buildings during 2023–2030, when 10%-50% of the electricity is provided by renewables. As shown in Fig. 11, each 10% increment of electricity provided by wind and solar energy can reduce 0.014–0.022 Mt CO_2_ in residential buildings and 0.004–0.009 Mt CO_2_ in public buildings.

**Figure 12:**
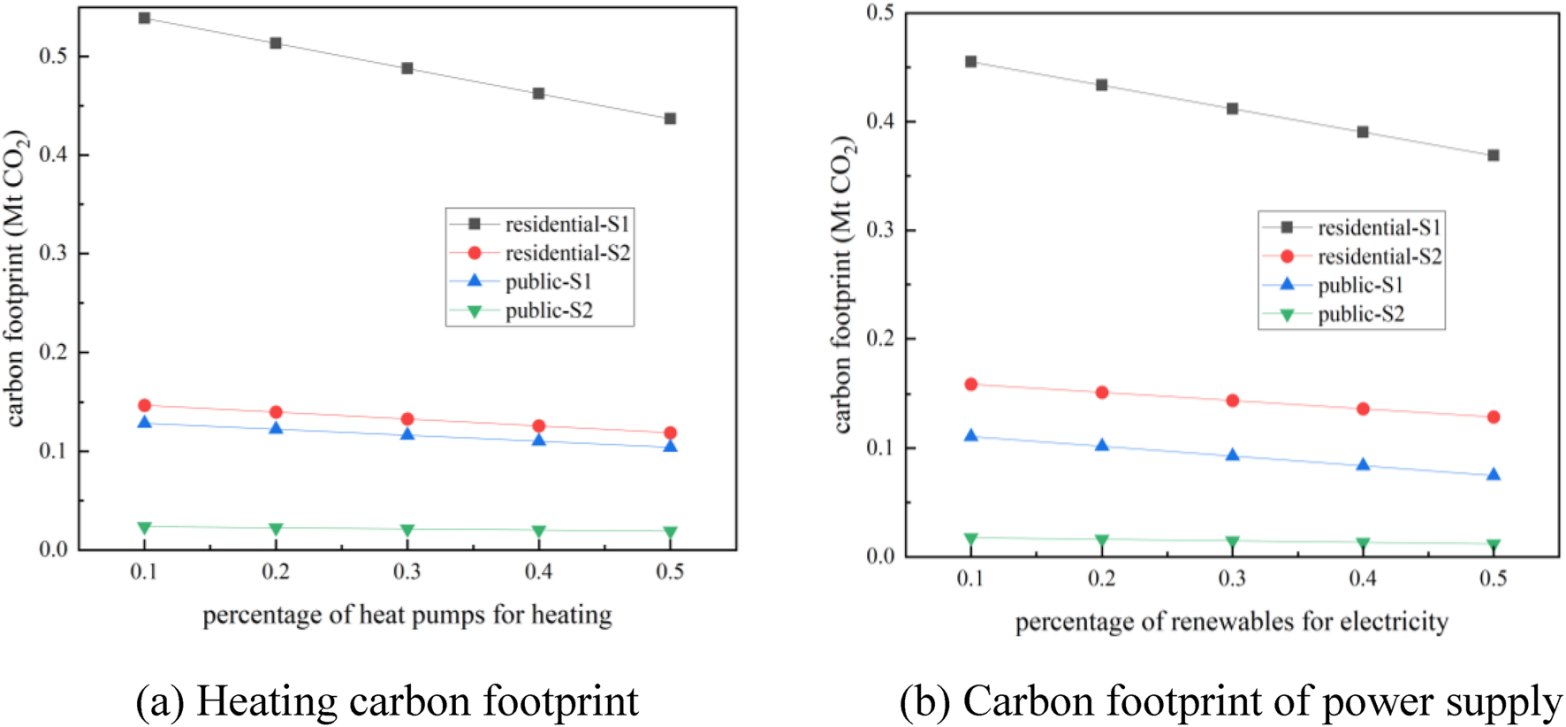
Carbon footprint under different floor area growth trends.

The faster the growth rate of building area is, the more obvious the rate of carbon footprint reduction will be. As shown in Fig. 12a, the reduction rate of heating carbon footprint of residential buildings under S1 trend is 3.7 times of that under S2 trend. The decrease rate of heating carbon footprint of public buildings under S1 trend is 5.4 folds of that under S2 trend. The use of renewable energy to supply electricity can significantly diminish the carbon footprint of buildings. As shown in Fig. 12b, the carbon footprint of electricity supply for residential buildings decreases 2.86 times faster under S1 trend than under S2 trend. The decrease rate of carbon footprint of electricity supply in public buildings under S1 trend is 6.3 times of that under S2 trend.

### Impact of the share of ultra-low energy buildings on energy consumption

**Figure 13:**
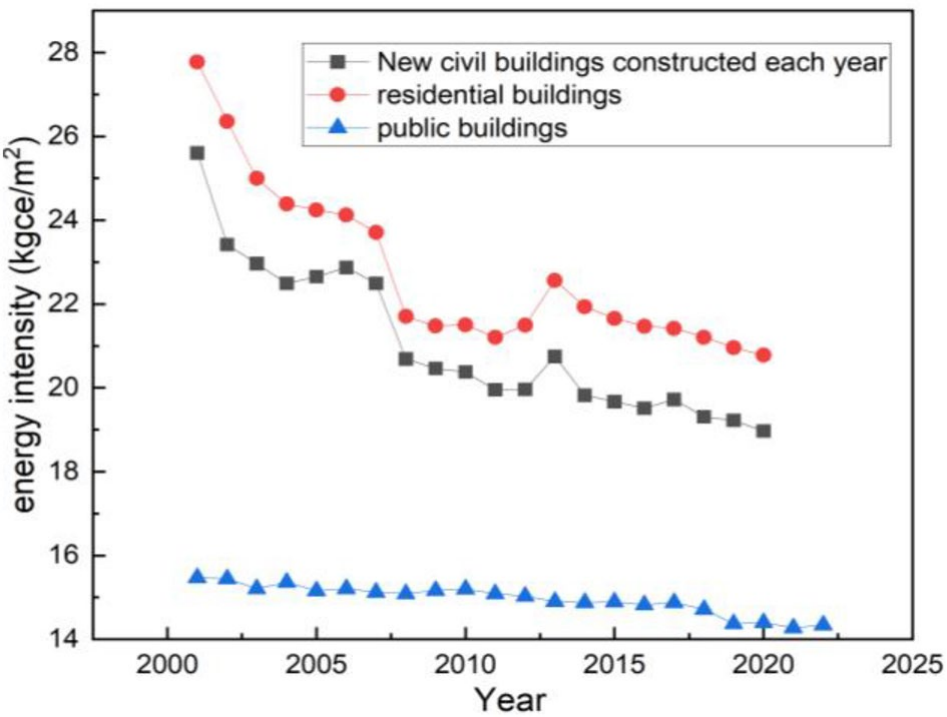
The energy intensity per unit floor area of buildings in Jilin Province.

The energy intensity per unit floor area (kgce/m^2^, kgce stands for kilograms of coal equivalent) of buildings in Jilin Province, which is extracted from Jilin Provincial Statistical Yearbook and Energy consumption standards for civil buildings of China, is shown in Fig. 13.

The energy intensity per unit of floor area of civil buildings declined from 25.61 to 19.95 kgce/m^2^ in 2001–2011, but showed a brief increase from 19.96 to 20.75 kgce/m^2^ in 2012–2013. After 2014, the energy intensity per unit of floor area has been on a decreasing trend to 18.97 kgce/m^2^ in 2020.

The energy intensity per unit of floor area of residential buildings has always been higher than that of civil buildings, and the trend is the same as that of civil buildings, from 27.78 kgce/m^2^ in 2001 to 20.78 kgce/m^2^ in 2020, with only a slight increase from 2011 to 2013. The energy intensity per unit floor area of public buildings has been showing a decreasing trend, from 15.47  to 14.34 kgce/m^2^.

The energy intensity of residential buildings is reduced to 11.6314 kgce/m^2^, when only the use of heat pumps and renewable energy is increased (by 10–50%) and the proportion of ultra-low energy buildings in new buildings is not taken into account.

**Figure 14:**
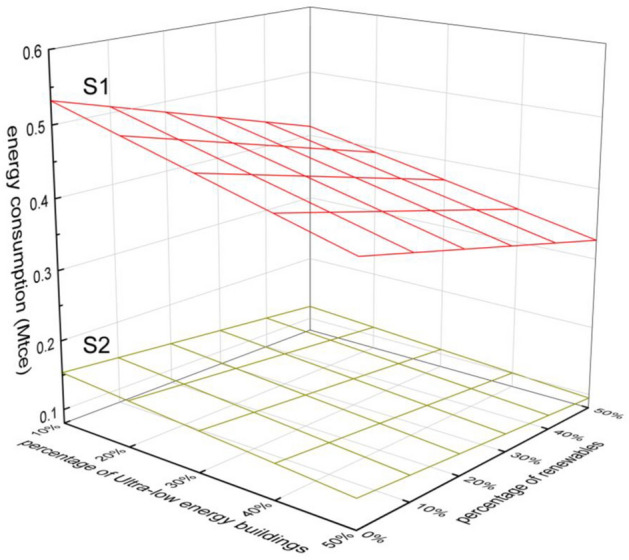
Impact of the share of ultra-low energy buildings on energy consumption.

The future new buildings are dominated by ultra-low energy buildings and near-zero energy buildings in order to save energy and reduce emissions. The impact of the proportion of ultra-low energy buildings on energy consumption in 2030 under different new construction floor area growth trends (S1 and S2) is shown in Fig. 14. When the new construction floor area develops in S1 trend, increasing the proportion of ultra-low energy buildings will accelerate the rate of decline of energy consumption in civil buildings as the proportion of renewable energy increases. Civil building energy consumption can be reduced from 0.53 Mtce to 0.33 Mtce in 2030 by improving clean energy utilization and increasing the proportion of ultra-low energy buildings. The faster the growth rate of building area is, the greater the impact of increasing the proportion of ultra-low energy buildings on building energy intensity will be.

When the proportion of ultra-low energy buildings is increased (by 10–50%), the energy intensity of civil buildings under S1 trend will be reduced to 8.7236 kgce/m^2^, and the energy intensity of civil buildings under S2 trend will be reduced to 9.8804 kgce/m^2^.

## Conclusions

The energy consumption and carbon footprint of buildings are profoundly influenced by variations in building area and the number of households. Therefore, it is imperative to forecast the growth trend of building area and number of households. This paper forecasts the number of residential households and the new civil building area in 2023–2030 by time series method. The prediction model is first validated by comparing the new construction area simulated by the SARIMA model with the actual new construction area. The new building area will show two trends of rapid growth (S1) and slow growth (S2) in the future. The energy consumption, heating energy carbon footprint, and non-heating energy carbon footprint of civil buildings (including residential and public buildings) are analyzed, and the effects of heat pump for heating, renewable (mainly solar and wind) power supply, and the share of ultra-low energy buildings are calculated as well. The following conclusions are obtained.

1. The time series method has a high accuracy and can be used for the prediction of future construction area changes by comparing the actual construction area with the predicted (or fitted) construction area from 2001 to 2022. Besides, the number of residential households will drop to 10.07 million.
2. The new building area in Jilin Province is expected to exhibit two trends of growth in the future: rapid growth (S1) and slow growth (S2). By 2030, under the S1 growth trend, the residential construction area and public building construction area in Jilin Province are expected to be 30.26 Mm^2^ (million square meters) and 7.23 Mm^2^, respectively. If the future floor area grows slowly under the S2 trend, the new floor area of different types will be 8.26 Mm^2^ and 1.33 Mm^2^ by 2030, respectively. The population growth shows a downward trend.
3. The faster the building area grows, the more the carbon footprint of building heating increases. The carbon footprint of civil buildings with coal as the main heating source will reach a maximum of 0.699 million tCO_2_ by 2030, which is 0.142 million tCO_2_ more than that of civil buildings with gas as the main heating source. The carbon footprint of non-heating energy in civil buildings is up to 0.596 million tCO_2_.
4. Increasing the proportion of renewable power supply can also achieve the effect of reducing the carbon footprint of buildings. The diminishing carbon footprint of buildings in the future can be achieved by controlling the growth rate of new building construction area and increasing the proportion of clean energy substitution. The carbon footprint is expected to be reduced by 0.017–0.311 million tCO_2_ when using heat pumps to supply 10–50% of the heat and wind and solar to supply 10–50% of the electricity.
5. Increasing the proportion of ultra-low energy buildings has a more obvious impact on the energy consumption of civil buildings than increasing the proportion of renewable energy sources. In the future, both retrofitting buildings with energy efficiency and improving the proportion of clean energy supply will significantly reduce building energy consumption, it is suggested to prioritize retrofitting buildings with energy efficiency before improving the proportion of clean energy supply. For every 10% increase in the use of ultra-low energy buildings, the energy consumption of civil buildings decreases in the range of 0.0063–0.028 Mtce. If the use of heat pumps and renewable energy increases by 10%, the energy consumption of civil buildings decreases in the range of 0.0054–0.0249 Mtce.

## Data Availability

The datasets generated during and/or analysed during the current study are available from the corresponding author on reasonable request.
